# Recognition of Common Non-Normal Walking Actions Based on Relief-F Feature Selection and Relief-Bagging-SVM

**DOI:** 10.3390/s20051447

**Published:** 2020-03-06

**Authors:** Pan Huang, Yanping Li, Xiaoyi Lv, Wen Chen, Shuxian Liu

**Affiliations:** College of Information Science and Engineering, Xinjiang University, Urumqi 830046, China; mrhuangpan@163.com (P.H.); xjuliyanping@163.com (Y.L.); xjuwawj01@163.com (X.L.); sd2020Chen@163.com (W.C.)

**Keywords:** Relief-F, relief-bagging-SVM, non-normal walking actions, feature selection, MEMS

## Abstract

Action recognition algorithms are widely used in the fields of medical health and pedestrian dead reckoning (PDR). The classification and recognition of non-normal walking actions and normal walking actions are very important for improving the accuracy of medical health indicators and PDR steps. Existing motion recognition algorithms focus on the recognition of normal walking actions, and the recognition of non-normal walking actions common to daily life is incomplete or inaccurate, resulting in a low overall recognition accuracy. This paper proposes a microelectromechanical system (MEMS) action recognition method based on Relief-F feature selection and relief-bagging-support vector machine (SVM). Feature selection using the Relief-F algorithm reduces the dimensions by 16 and reduces the optimization time by an average of 9.55 s. Experiments show that the improved algorithm for identifying non-normal walking actions has an accuracy of 96.63%; compared with Decision Tree (DT), it increased by 11.63%; compared with k-nearest neighbor (KNN), it increased by 26.62%; and compared with random forest (RF), it increased by 11.63%. The average Area Under Curve (AUC) of the improved algorithm improved by 0.1143 compared to KNN, by 0.0235 compared to DT, and by 0.04 compared to RF.

## 1. Introduction

With the development of microelectromechanical system (MEMS) technology and the popularization of smartphones with integrated acceleration sensors, action recognition is receiving increasing attention. It is widely used in medical health, pedestrian dead reckoning (PDR) and other fields [1,2]. In medical health, it is necessary to accurately measure the amount of exercise to provide more important reference indicators for clinical decision-making. In the PDR field, it is necessary to accurately predict the movement trajectory to determine the accurate position. The implementation of the above technical solutions is based on the accurate measurement of pedestrians. The number of steps is used as a basis. The current mainstream method for measuring the amount of exercise is to detect the number of steps taken by the patient, and the accuracy of the step counting is largely determined by motion recognition. The existing motion recognition algorithms have high accuracy for the detection of common normal steps. A combination of Global Positioning System (GPS) and MEMS technology can be used to identify uncommon movements, but weak indoor GPS signals are recognized facts in the technical field and difficult points to be solved. Therefore, existing methods do not have high accuracy in identifying abnormal walking postures, leading to lower accuracy of motion recognition and affecting the overall step counting accuracy. This article will explore the recognition of abnormal walking movements in depth.

There are two types of mainstream international methods of action recognition. The first category is the recognition and classification of actions based on human action video images. Wang, C. et al. [3] proposed a pose recognition method based on a neural network algorithm. This method uses an improved filtering algorithm to reduce the computational complexity of the scale invariant feature transforms (SIFT) operator and effectively reduce the impact on pose differences and image blur on the recognition rate. However, the recognition categories for non-normal walking actions are not comprehensive enough, resulting in a low overall recognition rate. Ince, O. F. et al. [4] proposed a new type of biometric recognition system for checking human activities in 3D space. The joint angle obtained by RGB depth sensors was used as a feature, and the average algorithm was used to reduce the dimension. This method has more advantages than other machine learning algorithms. The algorithm has a high recognition rate for faces for complex human motion recognition, but the recognition category for non-normal walking actions is not comprehensive enough. Ravbar et al. [5] used visual intuition to design an automatic behavior recognition system to identify behaviors in animals. This method can efficiently and accurately recognize the behavior and posture of various animals. The effect of this system on human motion recognition needs to be verified. Ullah et al. [6] proposed a framework of activity recognition using the surveillance video of industrial systems that used a convolutional neural network (CNN) to select shots based on human salient features and proposed multilayer long and short term memory. The results showed that this method is effective for identifying activities in an industrial environment. However, the incompleteness of common non-normal walking actions identified will have a certain impact on the overall recognition accuracy.

The second category is the recognition and classification of actions based on sensor signals. An et al. [7] proposed a 3D pedestrian position estimation algorithm for an inertial measurement unit (IMU) sensor worn on the waist, using a linear regression algorithm to improve the generalization ability of the classification algorithm for going downstairs and going upstairs. The subject of this article focuses on the study of normal walking movements, without considering the effects of abnormal walking actions such as writing and sleeping. Diete et al. [8] proposed an action recognition algorithm combining inertial sensors and interactive sensors. This algorithm recognized different human actions in different scenes by fusing data such as inertia and video. There is also the problem of insufficient analysis of abnormal walking movements. Kabir et al. [9] proposed a state-space-based linear activity recognition method. The model was very flexible in terms of control and used three real datasets to verify the validity of the model. In work by Kelly et al. [10], an action recognition method based on a genetic algorithm for sensor fusion was proposed. Support vector machines can be used to predict 10 different health indicators, and their errors do not exceed the clinical error benchmark. This article did not explore the impact on non-normal walking actions on health indicators. Lee, J.S et al. [11] used experimental heuristics to perform multi position PDR (mpPDR), which identified six poses and four patterns of carrying smartphones. Although the article explored the posture and position of normal walking movements in depth, the influence of non-normal walking actions on the experimental results was also very significant. Ma et al. [12] proposed an adaptive action recognition algorithm based on a time window, which solved the problem that different types of actions have different durations. Tong et al. [13] proposed a method of PDR using MEMS magnetic, angular velocity, and gravity sensors. This method reduced position drift caused by heading error and fault zero speed measurement. However, this method did not eliminate errors caused by non-normal walking actions.

Unfortunately, the current motion recognition algorithm is not comprehensive and has low recognition accuracy for common non-normal walking actions (such as sleep, irregular actions, writing, etc.), but the recognition of non-normal walking actions is of great significance to improve the reliability of medical and health indicators and the accuracy of PDR positioning. In order to fully and accurately identify non-normal walking actions, this paper proposes an MEMS action recognition method based on Relief-F feature selection and relief-bagging-SVM.

## 2. Data Acquisition and Preprocessing

**Figure 1:**
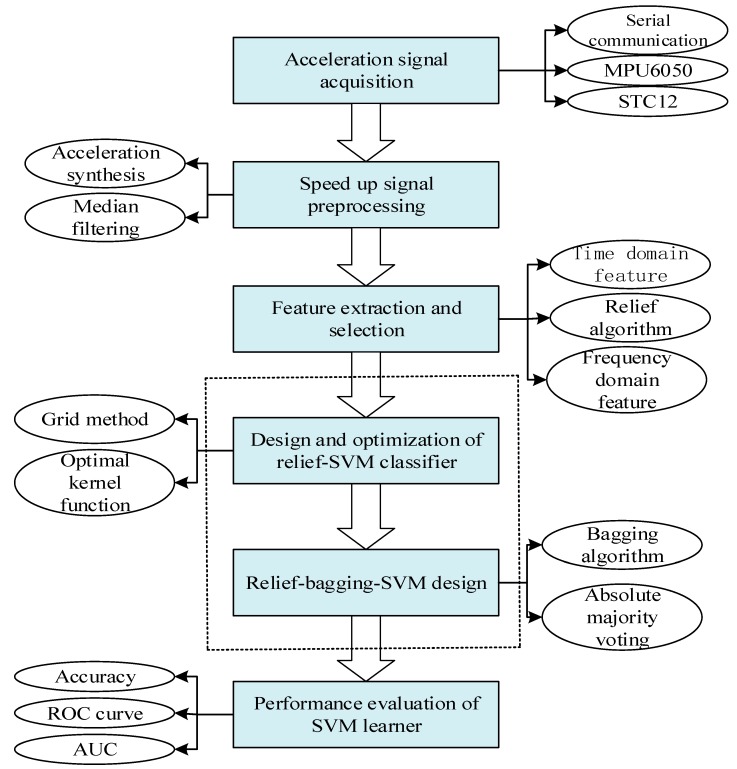
Framework of this article. Receiver Operating characteristic (ROC) Curve.

This paper uses an independently designed acceleration acquisition platform to obtain acceleration signals under different actions. After preprocessing, feature extraction and selection were performed, and the LG function was used for nonlinear normalization. The improved action recognition algorithm was used for identification and classification. The framework of this paper is shown in Figure 1.

### 2.1. Acceleration Acquisition Hardware Platform

**Figure 2:**
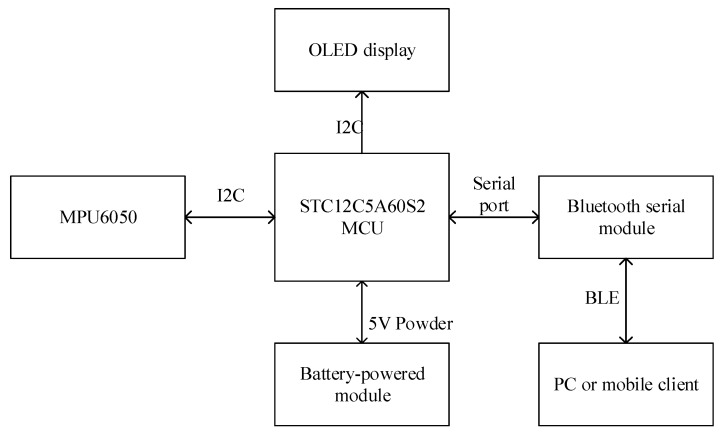
Hardware design diagram.

To obtain the acceleration information about human action accurately in real time, an acceleration signals acquisition device (worn on the wrist) was designed. The hardware design is shown in Figure 2 [14]. Some important parameter settings of the hardware platform are shown in Table 1.

### 2.2. Acceleration Signal Preprocessing

#### 2.2.1. Acceleration Synthesis

**Figure 3:**
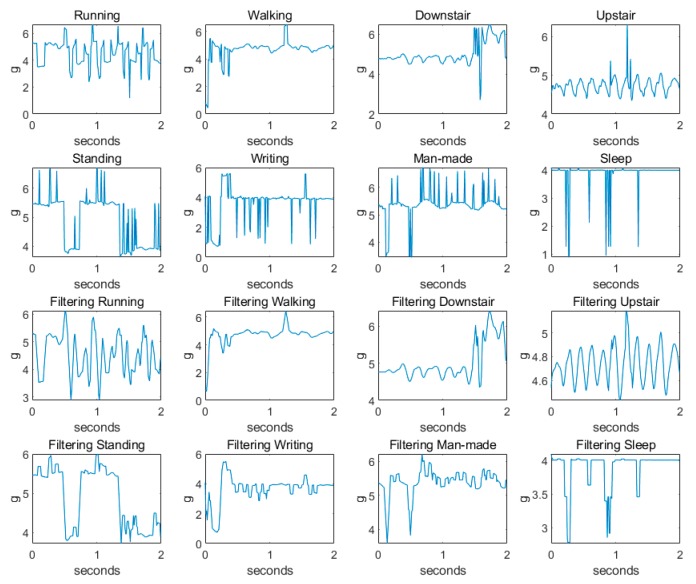
Acceleration signal after acceleration synthesis and filtering.

The randomness of the acceleration sensor carried by the human body causes the three-axis acceleration direction to be inconsistent with the direction of the human action. The noise generated by this factor can be partially eliminated by calculating the synthetic acceleration, as shown in Figure 3.

(1) $$a_{com}=\sqrt{a_{x}^{2}+a_{y}^{2}+a_{z}^{2}}$$

#### 2.2.2. Four-Point Median Filtering

Median filtering is a low-frequency enhanced spatial domain filtering technique. In this paper, the four-point median filtering principle is used to implement the linear filter [15]. The specific mathematical expression is shown in Equation (2). The processed synthetic acceleration signal is shown in Figure 3.

(2) $$\begin{array}{c} a_{avr}=Med\left\{a_{i}-v,\ldots ,a_{i}-1,a_{i}+1,\ldots ,a_{i}+v\right\}\\ i\in N,V=\frac{m-1}{2}\\ a_{avr}\left(n\right)=\overset{3}{\underset{i=0}{\sum }}a_{avr}\left(n-i\right)/4 \end{array}$$

From the analysis in Figure 3, it can be seen that the acceleration signals of the eight kinds of actions are subjected to acceleration synthesis and four-point median filtering to reduce the number of pseudo waves and glitches, which is more conducive to action recognition and classification.

## 3. Improvement of the Action Recognition Algorithm

### 3.1. Feature Extraction and Selection

Human action contains many characteristic parameters. Commonly used types include time-domain features, frequency domain features, wavelet transform features, and frequency domain features. This article uses the mean, standard deviation, variance, average slope, absolute slope, maximum, minimum, kurtosis, skewness, root mean square, interquartile range, correlation coefficient, and maximum in the time domain, fast Fourier transform (FFT) spectrum energy, FFT average power, and FFT median frequency. There are 15 features in total, and the feature vector has 23 dimensions so that the feature information of various actions can be relatively comprehensively extracted. However, these features are directly used for identification and classification. Due to the redundancy between these features and the complexity of the calculation, the final classification effect is not ideal. Therefore, this paper uses the relief algorithm to select features. By selecting features with significant classification weights, it can both improve the classification accuracy and reduce the time complexity of the algorithm. The features extracted in this paper are shown in Table 2.

The relief algorithm is a commonly used filtering feature selection algorithm. This algorithm mainly designs a relevant statistic to measure the importance of features. In this paper, the relevant statistic is defined as the feature classification weight, as shown in Equation (3).

(3) $$\sigma^{k}=\underset{i}{\sum }-diff{\left(x_{i}^{k},x_{i,nh}^{k}\right)}^{2}+diff{\left(x_{i}^{k},x_{i,nm}^{k}\right)}^{2}$$

The acceleration signals of eight kinds of actions are extracted from this paper, and the support vector machine with eight classifications is used. Therefore, relief is deformed, and Relief-F is used to solve the eight classification problems in this paper [16,17].

(4) $$\sigma^{k}=\underset{i}{\sum }-diff{\left(x_{i}^{k},x_{i,nh}^{k}\right)}^{2}+\underset{l\neq w}{\sum }\;\left(p_{l}\times diff{\left(x_{i}^{k},x_{i,nm}^{k}\right)}^{2}\right)$$

Among them, $x_{i,nh}^{k}$ in Equations (3) and (4) is “near-hit” and $x_{i,nm}^{k}$ is “near-miss”, $\;x_{i}^{k}$ represents the value of sample $x_{i}$ on feature k, and $diff{\left(x_{i}^{k},x_{i,nh}^{k}\right)}^{2}$ determines the type of feature k. $p_{l}$ is the proportion of type l samples of the dataset.

**Figure 4:**
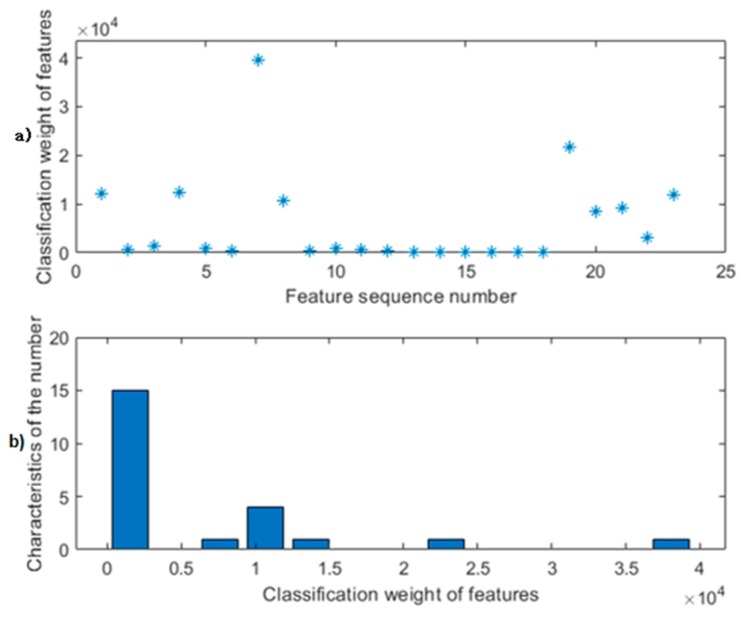
Feature classification weight diagram before threshold setting: (**a**) Upper submap: Feature weight value map; (**b**) Lower submap: Weight value frequency chart.

**Figure 5:**
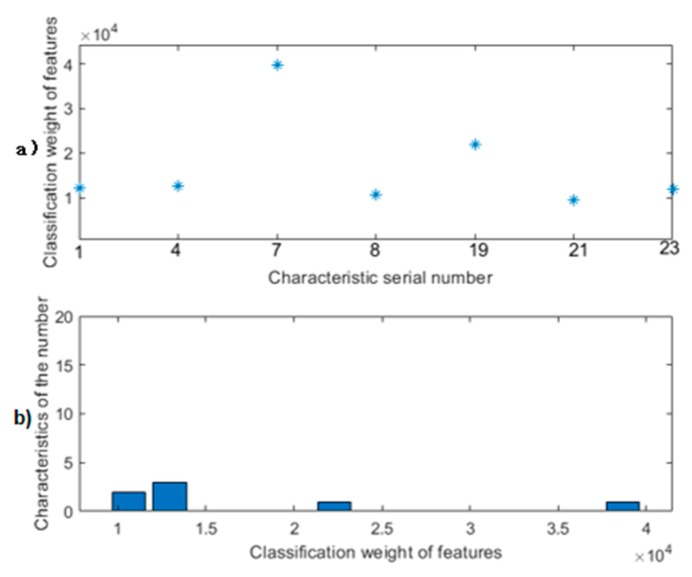
Feature classification weight diagram after threshold setting. (**a**) Upper submap: Feature weight value map; (**b**) Lower submap: Weight value frequency chart.

The feature classification weights map analyzed by relief after feature extraction is shown in Figure 4. After analyzing the observation data onto a large number of experiments, setting the classification weights threshold to 9000 is the best feature selection effect, and continuous fusion of the selected seven-dimensional features is shown in Figure 5 to achieve the best classification effect. The serial number of the seven-dimensional feature is good. The feature names are shown in Table 3.

**Figure 6:**
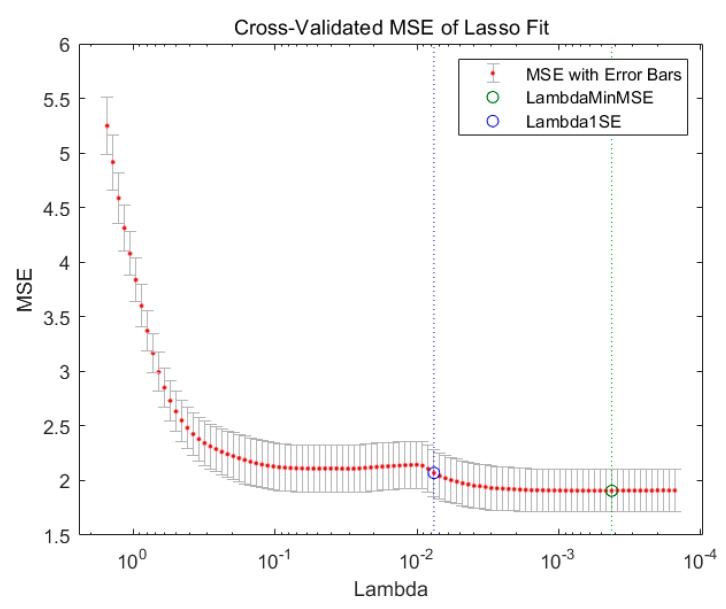
10-fold cross-validation of polynomial kernel optimization.

In this paper, the mainstream feature selection method least absolute shrinkage and selection operator (LASSO) and principal component analysis (PCA) are compared horizontally. The classification effect of (LASSO) is shown in Figure 6. The classification accuracy is shown in Table 4.

After comprehensive analysis of Table 4, Figure 4, Figure 5 and Figure 6, the average accuracy of the Relief-F method is 30.83% higher than the LASSO method, far better than LASSO only methods.

### 3.2. Design and Optimization of Relief-SVM

SVM is one of the mainstream algorithms for statistical machine learning. SVM has an excellent effect on the classification of small sample data. SVM can map linearly indivisible samples to the hyperplane for classification, and its essence is to solve the smallest problem. The minimum soft interval problem from the support vector to the dividing line is shown in Equation (5). This is a convex quadratic programming problem. In the face of the quadratic programming problem with constraints, this paper uses the Lagrange operator method, which is then transformed into solving the dual problem, as shown in Equation (6) [18,19,20].

(5) minw,b12∥w∥2+C∑i=1mι01(y(wTxi+b)−1). s01={1,(y(wTxi+b)−1)<00,(y(wTxi+b)−1)≥0

(6) $$max_{\alpha }=\overset{m}{\underset{i}{\sum }}\alpha_{i}-\frac{1}{2}\overset{m}{\underset{i}{\sum }}\overset{m}{\underset{j}{\sum }}\alpha_{i}\alpha_{j}y_{i}y_{j}x_{i}^{T}x_{j}.\;s.t.\;\overset{m}{\underset{i}{\sum }}\alpha_{i}y_{i}=0,0\leq \alpha_{i}\leq C$$

**Figure 7:**
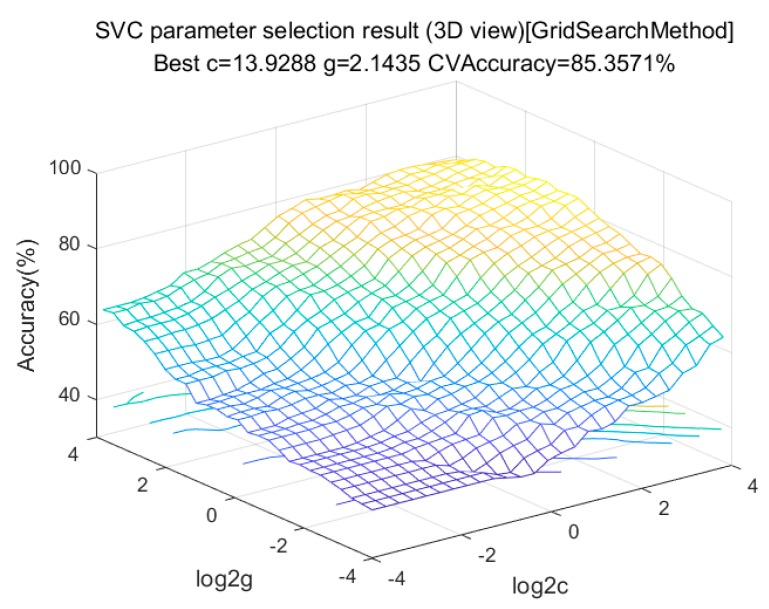
10-fold cross-validation of polynomial kernel optimization.

ι01 in Equation (5) is a loss function. Since the theoretical loss function has noncontinuous and nonconvex mathematical properties that are difficult to solve, a function that is easy to solve is used instead. In Equation (6), C represents a penalty coefficient, which determines the number of support vectors and is one of the key indicators. The complexity of the inner product of $x_{i}^{T}x_{j}$ determines the performance of the SVM to a large extent, so the sample can be mapped to an optimal high-dimensional space by using an appropriate kernel function and gamma value. Therefore, this paper uses the grid optimization method and k-fold cross-validation method to solve the optimal C and gamma, as shown in Figure 7, and the classification kernel to select the most suitable kernel function is shown in Table 5.

It can be seen in Table 5 that the optimal kernel function is a polynomial kernel, and the optimal penalty coefficient C corresponding to the polynomial kernel is 13.9288, and the optimal gamma value is 2.1435.

### 3.3. Design of Relief-Bagging-SVM

**Figure 8:**
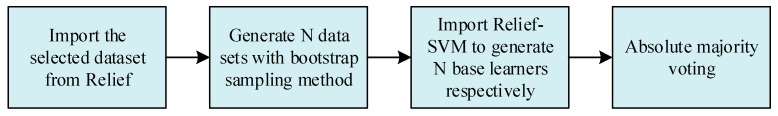
Relief-bagging-SVM algorithm flow.

From the analysis in Table 6 and Table 7, it can be seen that although Relief-SVM has excellent performance in time performance and classification accuracy, the classification accuracy in man-made step counting is only 40%. The focus and innovation of this article are on the recognition and classification of abnormal walking actions. Ensemble learning performs well in improving the classification performance of weak learners. Therefore, the bagging algorithm in ensemble learning is used to design a relief-bagging-SVM action recognition algorithm, as shown in Figure 8, to accurately identify man-made step counting and other abnormal walking actions.

The key to the performance of relief-bagging-SVM is to generate “good and different” base learners. In this paper, bootstrap sampling is used to generate different datasets. The bootstrap sampling method is for a dataset that includes M samples. According to the principle of non-replacement extraction, sampling is performed M times to obtain a training set, and according to the analysis of Equation (7), it can be seen that each training set includes only 63.2% of the original data, while the remaining 36.8% of the samples are used as the test set [21].

(7) $$lim_{M\to \propto }{\left(1-\frac{1}{M}\right)}^{M}=\frac{1}{e}\approx 0.368$$

$\frac{1}{M}$ in Equation (7) is the probability that each sample is taken.

### 3.4. Performance Evaluation of SVM Learners

This article uses the Receiver Operating Characteristic curve (ROC) and AUC to evaluate the modeling effect of the SVM learner. The samples are sorted according to the score of the predictor’s prediction result, the samples are predicted as positive examples in this order, the true positive rate (TPR) is calculated each time as Equation (8), and the false positive (false positive rate, FPR) is shown in Equation (9), where TPR is the vertical axis of the ROC curve and FPR is the horizontal axis of the ROC curve.

(8) $$TPR=\frac{TP}{TP+FN}$$

(9) $$FPR=\frac{FP}{TN+FP}$$

In Equation (8), TP, FN, FP, and TN in Equation (9) are the true positive examples, false negative examples, false positive examples, and true negative examples in the classification result confusion matrix, respectively.

## 4. Experiments and Results Analysis

The experimental platform of this article uses Matlab2018a. By observing the daily life of a large number of students and faculty members of Xinjiang University and sampling with a questionnaire survey, we found that the top four most frequent occurrences of non-normal walking actions are: standing (defined as people who have no regular or obvious movements, with a few occasional movements; for example, sitting would be considered a standing posture in this article), writing (defined as the state of a person writing with a pen or chalk), sleeping, and man-made (this action exists more in China, especially in some college students; in order to increase the number of steps in various apps, various devices are used to simulate the movement of the human body to create the number of steps. The acceleration trajectory of such devices is a sine or cosine diagram). These four non-normal walking actions account for more than 90% of all non-normal walking actions, so this article focuses on the four types of non-normal walking actions. The first four occurrences of normal walking actions are: walking, upstairs, downstairs, and running. These four occupy 99% of the normal walking actions of the human body. To reduce the effect of age, height, weight, and gender on the robustness of the algorithm, our experiment object consisted of four men and four women, aged between 19–45 years old, weighing between 45–85 kg with a height between 150–190 cm. The experimental data collect acceleration information of eight kinds of actions in Table 8. For each walking action, 5000 synthetic acceleration information is collected, which takes 50 s and is processed by frame. Each sample generates 50 samples, so the experimental acceleration of the dataset has a total of 400 samples (acceleration information for eight different ages, heights, weights and genders under each action,). After the relief feature selection, the features are serially fused, and the dataset becomes a 400 × 7 feature matrix. The experimental data used a bootstrap sampling method to generate a training set and a test set. Cross-validation was used during the experiment. The 10-fold cross-validation method [22,23] uses the libsvm package to implement SVM multiclassification [24]. From the analysis above, we know that the polynomial kernel is used for experiments. The number of basic learners for integrated learning is five, as shown in Table 6 and Table 7. The average is taken after 10 category experiments. This paper compares the results with mainstream decision tree (DT), k-nearest neighbor (KNN) and random forest (RF). The classification accuracy is shown in Table 6. The time performance is shown in Table 7. DT, RF and KNN parameter adjustments are shown in Table 9.

### 4.1. Action Classification Accuracy and Time Performance

It can be intuitively seen from Table 6 that the traditional methods for identifying non-normal walking actions are not effective, and the improved algorithm for identifying non-normal walking actions had an accuracy of 96.63%. Compared with DT, it increased by 11.63%; compared with KNN, it increased by 26.62%; and the recognition performance has been greatly improved.

From Table 6 and Table 7, the average classification accuracy of the improved relief-bagging-SVM reached 94.37%. Compared with relief-SVM, the average classification accuracy improved by 2.71% while maintaining time performance. The accuracy of step counting reached 100%, which increased by 60%. Compared with SVM, both the time performance and classification accuracy were comprehensively and greatly improved. The average recognition accuracy of the improved algorithm was 9.37% higher than KNN, 1.87% higher than DT and 1.87% higher than RF.

### 4.2. ROC Curve of the SVM Model

**Figure 9:**
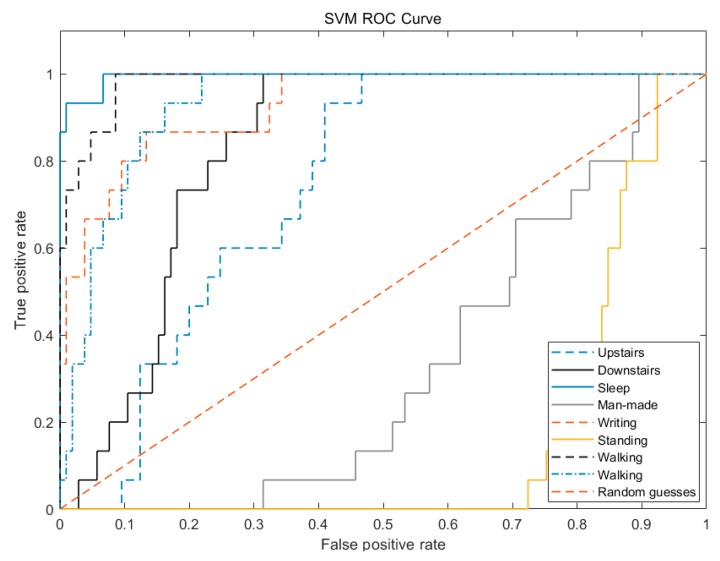
SVM ROC curve.

In Figure 9, the man-made step counting and standing ROC curves of the SVM are both below the random guess curve. The recognition and classification of these two types are worse than the random guess, and the ROC curves of the other six actions are in the random guess model. Above, the properties of the ROC curve are generally average.

**Figure 10:**
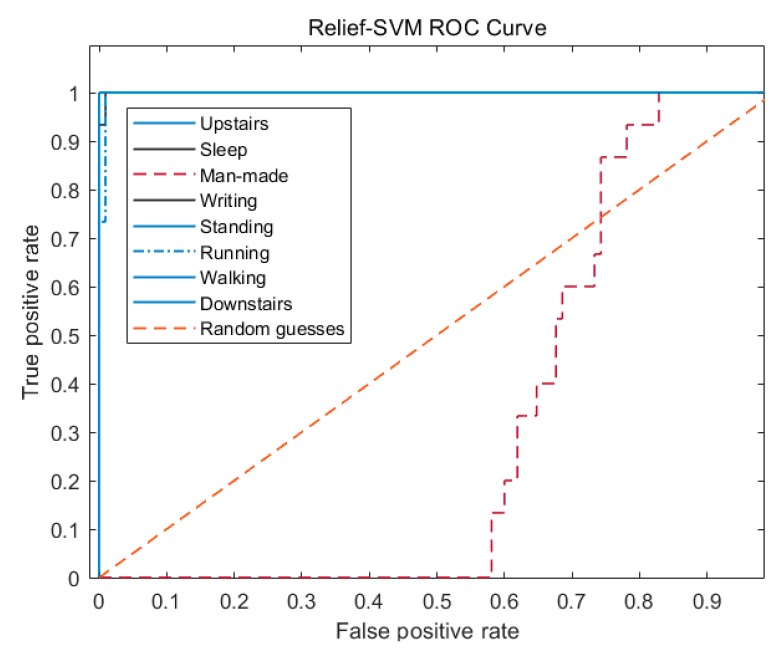
Relief-SVM ROC curve.

In Figure 10, the man-made step counting ROC curve of relief-SVM is much lower than the random guess curve, and its classification recognition effect is not ideal. However, the ROC curves of the other seven categories have good properties, and the classification effect and generalization ability are very strong.

**Figure 11:**
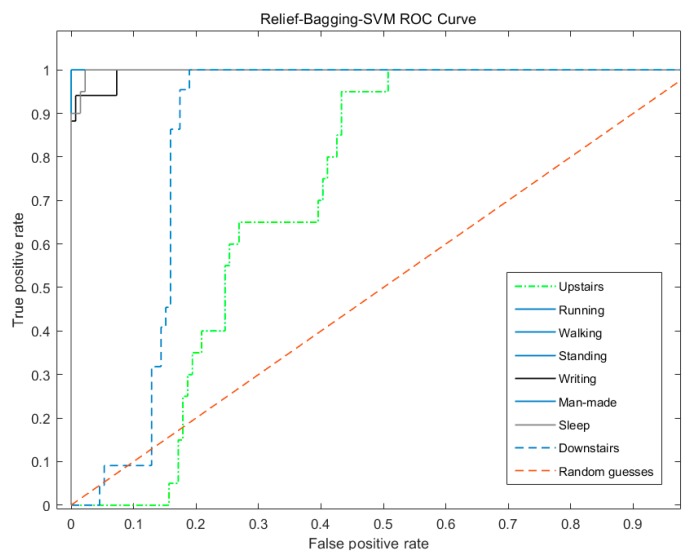
Relief-bagging-SVM ROC curve.

In Figure 11, the properties of the ROC curve of the man-made step counting of relief-bagging-SVM have reached the optimal, and except for the properties of the ROC curve of the upstairs and the downstairs, are worse compared with the relief-SVM.

From Table 10 combined with the analysis of Figure 9, Figure 10 and Figure 11, it can be seen that during abnormal walking actions, the AUC of relief-bagging-SVM improved compared to SVM and relief-SVM. The average AUC of relief-bagging-SVM reached 0.9466, a 0.0325 improvement over the relief-SVM, and a 0.2089 improvement over the SVM. Comprehensive analysis showed that the generalized ability and time performance of the improved relief-bagging-SVM model were the best.

**Figure 12:**
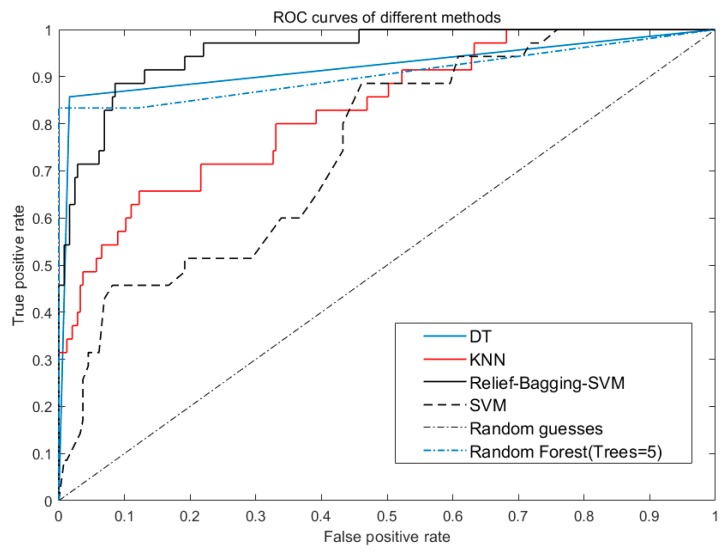
ROC curves of SVM, KNN, DT and Relief-Bagging-SVM.

From Table 10 and Figure 12, it can be concluded that the average AUC of the improved algorithm improved by 0.1143 compared to KNN, 0.0235 compared to DT and 0.04 compared to RF.

### 4.3. Leave-One-Person-Out Cross Validation

We also used Leave-One-Person-Out cross validation (LOPOCV) to evaluate the generalization ability of our model, and the basic situation of eight test subjects is shown in Table 8. The test results are shown in Table 11.

In Table 8, we have listed in detail the personal information of the eight people who participated in the experiment.

We used seven people’s data for training each time, leaving one person’s data for testing. The test results are shown in Table 8. The average accuracy of the eight people was 94.25%, and the variance was 0.62%. The standard deviation was 0.79%.

## 5. Conclusions

This paper explores common non-normal walking actions recognition problems and solves the problems of incomplete classification and low accuracy of the existing motion recognition algorithms for non-normal walking actions. It uses the relief algorithm for feature selection and the improved relief-bagging-SVM algorithm for action classification. The improved algorithm for identifying non-normal walking actions has an accuracy of 96.63%. Compared with decision tree (DT), it increased by 11.63%; compared with k-nearest neighbor (KNN), it increased by 26.62%, the average AUC of the improved algorithm improved by 0.1143 compared to KNN, improved by 0.0235 compared to DT, improved 0.04 compared to RF. These results are of great practical significance to the accuracy of medical health indicators and the detection of steps in PDR.

## Figures and Tables

**Table 1:** Some important parameter settings of the hardware platform.

Hardware	Parameter	Value	Parameter	Value
MPU6050	Sampling frequency	125 Hz	acceleration sensitivity/LSB/g	16,384
	Sampling range	−4 g~4 g	-	-
STC12 MCU	*x*-axis acceleration register	0 × 3B, 0 × 3C	*y*-axis acceleration registers	0 × 3D, 0 × 3E
	*z*-axis acceleration registers	0 × 3F, 0 × 40	-	-
BLE	Communication cycle	10 ms	Baud rate/bps	11,520
	Way of communication	Asynchronous serial	-	-

**Table 2:** Feature name and corresponding dimension table. FFT: Fast Fourier transform.

Category	Feature Name	Dimension	Feature Name	Dimension
	mean	1	absolute slope	9
	standard deviation	1	maximum	1
Time Domain	skewness	1	root mean square	1
	correlation coefficient	1	maximal value	1
	variance	1	minimum	1
	average slope	1	kurtosis	1
FFT	spectrum energy	1	average power	1
	median frequency	1	-	-

**Table 3:** Feature number and name after Relief-F selection.

Number	Feature Name	Number	Feature Name	Number	Feature Name
1	mean	8	average slope	23	median frequency
4	root mean square	19	maximum	-	-
7	maximal value	21	skewness	-	-

**Table 4:** Classification effect of different feature selection methods (Polynomial). LASSO: least absolute shrinkage and selection operator.

Category	LASSO-SVM	Relief-F-SVM
Walking (%)	80.00	100.0
Running (%)	93.33	100.0
Standing (%)	13.33	100.0
Writing (%)	86.66	93.33
Man-made (%)	13.33	40.00
Sleep (%)	93.33	100.0
Downstairs (%)	40.00	100.0
Upstairs (%)	66.66	100.0
Average accuracy (%)	60.83	91.66

**Table 5:** Relief-F-SVM kernel function classification accuracy.

Category	Linear	Polynomial	Gauss	Sigmoid
Walking (%)	100.0	100.0	93.33	0
Running (%)	100.0	100.0	100.0	0
Standing (%)	93.33	100.0	93.33	0
Writing (%)	93.33	93.33	93.33	100.0
Man-made (%)	33.33	40.00	26.66	0
Sleep (%)	86.67	100.0	86.66	0
Downstairs (%)	93.33	100.0	93.33	0
Upstairs (%)	53.33	100.0	100.0	12.50
Average accuracy (%)	81.66	91.66	85.83	12.50

**Table 6:** Action recognition and classification accuracy.

Category	KNN	RF	DT	SVM	Relief-SVM	Relief-Bagging-SVM
Walking (%)	100.0	93.75	100.0	80.00	100.0	100.0
Running (%)	100.0	100.0	100.0	93.33	100.0	100.0
Standing (%)	73.33	91.67	100.0	13.33	100.0	94.87
Writing (%)	86.66	38.46	100.0	86.67	93.33	94.12
Man-made (%)	40.00	100.0	40.00	13.33	40.00	100.0
Sleep (%)	80.00	85.71	100.0	93.33	100.0	97.56
Downstairs (%)	100.0	100.0	100.0	40.00	100.0	86.84
Upstairs (%)	100.0	94.74	100.0	66.67	100.0	81.58
Normal Actions (%)	100.0	97.12	100.0	70.00	100.0	92.10
Non-normal Actions (%)	69.99	78.96	85.00	51.66	83.33	96.63
Average accuracy (%)	85.00	88.04	92.50	60.83	91.66	94.37

**Table 7:** Time performance table.

Time/Dimension	KNN	RF	DT	SVM	Relief-SVM	Relief-Bagging-SVM
Feature dimension	23	23	23	23	7	7
Search time(s)	1.36	0.58	0.81	18.68	9.13	9.13

**Table 8:** Basic table of laboratory personnel.

People	1	2	3	4	5	6	7	8
Sex	Man	Woman	Woman	Man	Woman	Man	Woman	Man
Weight/kg	63	51	45	85	56	80	55	70
Height/cm	170	165	157	183	161	191	155	178
Age	26	21	19	45	42	39	23	22

**Table 9:** Some important parameters of k-Nearest Neighbor (KNN), Decision Tree (DT), Random Forest (RF) and SVM.

Classifier	Parameter	Value	Parameter	Value
DT	Maximum number of splits	100	Surrogate decision splits	off
	Split criterion	Cini’s diversity index	Maximum surrogates per node	10
SVM	Kernel function	Polynomial	Kernel scale mode	Manual
	Multiclass method	One-vs-One	Standardize data	on
RF	Pruning strategy	Post-pruning	Tree numbers	5
	Split criterion	Cini’s diversity index	Max features	23
KNN	Number of neighbors	1	Distance weight	Equal
	Distance metric	Euclidean	Standardize data	on

**Table 10:** AUC table.

Category	RF	KNN	DT	SVM	Relief-SVM	Relief-Bagging-SVM
Walking	-	-	-	0.9321	1	1
Running	-	-	-	0.9822	0.9975	1
Standing	-	-	-	0.1562	1	1
Writing	-	-	-	0.9283	0.9994	0.9953
Man-made	-	-	-	0.3321	0.3162	1
Sleep	-	-	-	0.9949	0.9994	0.9981
Downstairs	-	-	-	0.8317	1	0.8567
Upstairs	-	-	-	0.7441	1	0.7231
Average AUC	0.9066	0.8323	0.9231	0.7377	0.9141	0.9466

**Table 11:** Leave-One-Person-Out cross validation (LOPO-CV) experiment accuracy table.

Category	1	2	3	4	5	6	7	8
Walking (%)	100.0	98.32	97.63	100.0	98.66	100.0	97.22	100.0
Running (%)	100.0	100.0	100.0	100.0	100.0	100.0	100.0	100.0
Standing (%)	95.87	93.61	95.11	94.33	97.65	94.18	97.58	96.33
Writing (%)	93.12	93.21	94.64	93.21	92.47	95.66	97.55	98.11
Man-made (%)	100.0	96.21	93.22	94.31	92.10	95.33	93.11	94.56
Sleep (%)	95.56	96.55	96.12	94.33	96.58	93.22	95.12	94.23
Downstairs (%)	90.84	88.63	84.25	92.31	97.46	95.21	86.33	89.94
Upstairs (%)	80.58	84.16	82.15	86.21	87.68	80.14	83.22	88.21
Average accuracy (%)	94.49	93.83	92.89	94.33	95.32	94.21	93.76	95.17
